# Proton Reduction with a Cobalt-Doped Thiomolybdate Cluster: A Structural and Functional Model of Co-Doped MoS_2_

**DOI:** 10.1021/jacs.5c09888

**Published:** 2025-10-15

**Authors:** Kamaless Patra, Leyla R. Valerio, Zhou Lu, Kaye Kuphal, William W. Brennessel, Ellen M. Matson

**Affiliations:** Department of Chemistry, 6927 University of Rochester, Rochester, New York 14627, United States

## Abstract

A heterometallic thiomolybdate cluster, Cp*_3_CoMo_2_S_4_ (Cp* = 1,2,3,4,5-pentamethylcyclopentadienide) has been synthesized and identified as a molecular electrocatalyst for proton reduction in dimethylformamide. Compared with its homometallic congener (Cp*_3_Mo_3_S_4_), cobalt incorporation improves activity by lowering the overpotential for proton reduction, consistent with the contrasting catalytic performance of MoS_2_ and its Co-doped derivative. Isolation of the reduced form of Cp*_3_CoMo_2_S_4_ and subsequent reactivity studies provide insight into the reaction pathway. These findings establish Cp*_3_CoMo_2_S_4_ as a molecular model for extended sulfide materials.

## Graphical abstract

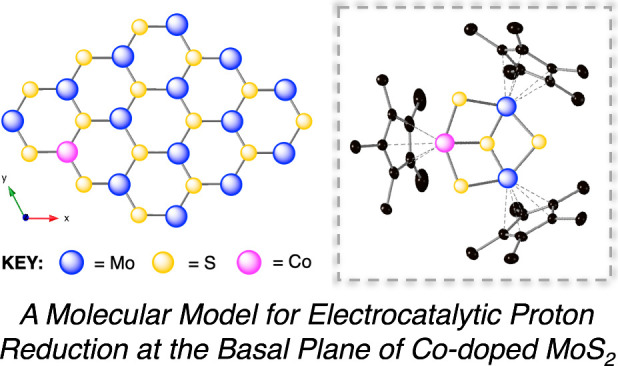

Electrocatalytic hydrogen evolution reaction (HER) is an important process in energy conversion and storage technologies.^1,2^ One class of electrocatalysts for HER are layered molybdenum disulfides (MoS_2_).^3–5^ These materials are attractive due to their abundance, low cost, and potential for energy conversion applications.^6^ However, catalytically active sites are found only along the edges of MoS_2_ layers, rendering large portions of the 2D material inactive for proton reduction.^5,7–10^ One approach to circumvent this deficiency involves the formation of MoS_2_ nanoparticles that possess increased exposure of the active edge.^11–13^ Alternatively, the installation of transition metal dopants (e.g., Co, Zn, Pd) along the basal plane of MoS_2_ has been credited with activating the surface for HER.^14–16^

Given the significance of the discovery that elemental dopants increase HER at the basal plane of MoS_2_, it is important to understand the role the heteroatom plays in mediating catalysis. Thiomolybdate clusters represent simplified structural models of the surface of MoS_2_, allowing atomistic insight into reactivity and mechanisms of small molecule activation. The majority of thiomolybdate clusters reported to date possess high sulfur to metal ratios, rendering them excellent models for the *edge* sites of MoS_2_.^17^ Consistent with the material itself, the aforementioned thiomolybdate clusters have been reported as homogeneous electrocatalysts for HER. To model the *basal plane* of MoS_2_, researchers have turned to a series of cuboidal trinuclear thiomolybdate assemblies with the general formula “L_3_Mo_3_S_4_^n^”.^17^ Indeed, versions of these assemblies catalyze small molecule hydrogenation chemistries.^18–20^ However, to date, limited examples of heterometal-doped hemicuboidal thiomolybdate clusters have been reported,^21–25^ with no studies detailing the reactivity of these systems.

We became interested in generating hemicuboidal clusters, with the general formula L_3_CoMo_2_S_4_^n^, as models of the basal plane of Co-doped MoS_2_. We hypothesized the use of an ancillary ligand on the heterometallic precursor would favor the addition of a single dopant to the thiomolybdate dimer. The preparation of the Co-doped thiomolybdate, Cp*_3_CoMo_2_S_4_, was accomplished as outlined in Scheme 1 (see SI for details).

**Scheme 1:**
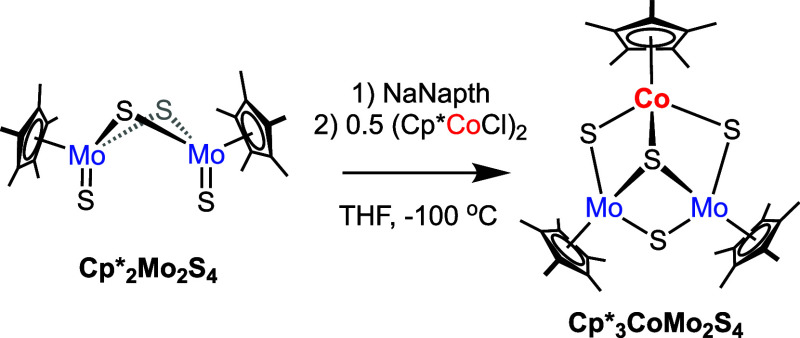
Synthesis of Cp*_3_CoMo_2_S_4_

Confirmation of product formation was obtained through single-crystal X-ray diffraction. Refinement of the data reveals integration of the cobalt center into the hemicuboidal assembly at a molybdenum site (Figure 1, Tables S1–S2). Comparison of the structure of Cp*_3_CoMo_2_S_4_ and its homometallic congener, Cp*_3_Mo_3_S_4_,^26^ provides an opportunity to assess the consequences of Co substitution (Table S2). The average Mo–(μ_2_-S) bond length in Cp*_3_CoMo_2_S_4_ is 2.296 Å, shorter than those reported for Cp*_3_Mo_3_S_4_ (2.309 Å). Shortening of Mo–S bond distances is consistent with oxidation of molybdenum (e.g., Mo–S bond length in [Cp*_3_Mo_3_S_4_]^+^ = 2.297 Å).^27^ Similarly, the Mo–Mo distance in Cp*_3_CoMo_2_S_4_ (2.8339(3) Å) resembles that of the oxidized homometallic assembly (Cp*_3_Mo_3_S_4_ = 2.861(6) Å;^26^ [Cp*_3_Mo_3_S_4_]^+^ = 2.819(5) Å^27^). Taken together, these observations indicate that doping with a low-valent cobalt center results in the increase in the aggregate oxidation states of the host metals. The average Co–(μ_2_-S) bond length is 2.296 Å, consistent with literature-reported Co­(II)–(μ_2_-S) distances.^28,29^ In total, bond metric analysis suggests that the oxidation state distribution of metals in Cp*_3_CoMo_2_S_4_ includes Co­(II), Mo­(V) and Mo­(IV) centers. However, definitive assignment of oxidation states is the subject of ongoing investigations.

**Figure 1:**
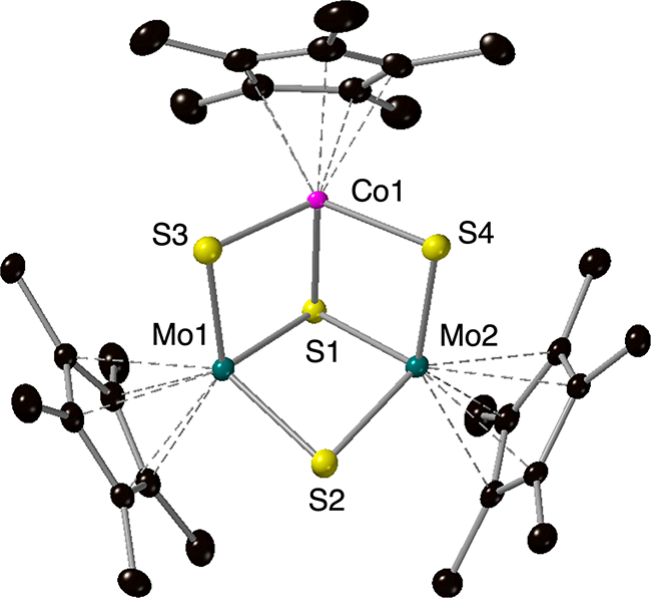
Molecular structure of Cp*_3_CoMo_2_S_4_ shown with 50% probability ellipsoids. Hydrogen atoms have been removed for clarity. Key: C, black; S, yellow; Co, pink; Mo, teal.

To understand the electrochemical consequences of Co-doping within the thiomolybdate assembly, the cyclic voltammogram (CV) of Cp*_3_CoMo_2_S_4_ is compared with that of Cp*_3_Mo_3_S_4_ (Figure 2, Table S3). Cp*_3_Mo_3_S_4_ displays two reversible redox events at *E*_1/2_ = −1.23 V ([Cp*_3_Mo_3_S_4_]^+/0^) and −2.22 V ([Cp*_3_Mo_3_S_4_]^0/–^) vs Fc/Fc^+^ in dimethylformamide (DMF; 0.1 M TBAPF_6_ used as the supporting electrolyte). In contrast, the CV of Cp*_3_CoMo_2_S_4_ exhibits three reversible redox events located at *E*_1/2_ = −1.80 V, −0.96 V and −0.252 V. It is noted that density of state calculations on Co-doped MoS_2_ have indicated that the dopant introduces midgap states with minor contributions from Mo and S atoms.^30,31^ This change is mirrored by the observation of an additional redox event in the CV of Cp*_3_CoMo_2_S_4_.

**Figure 2:**
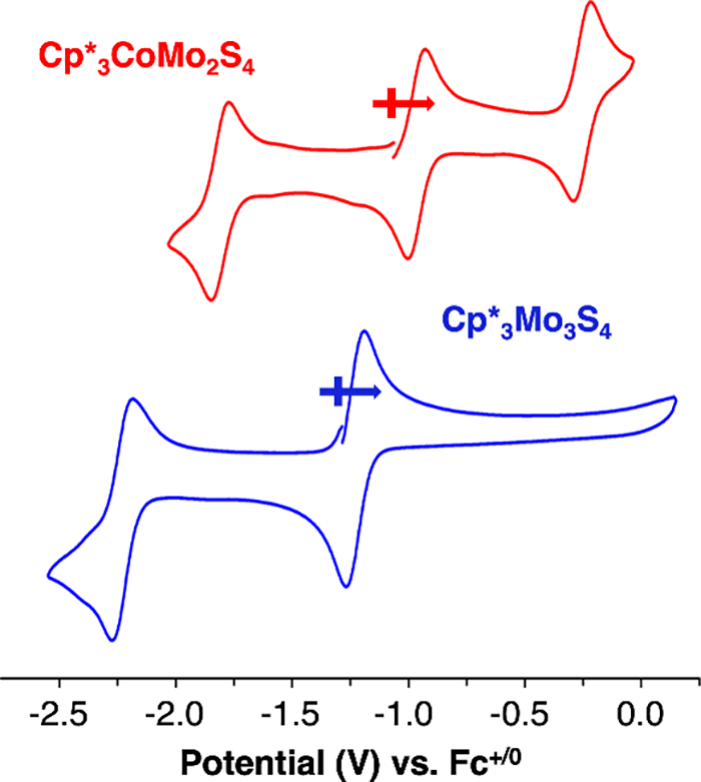
CV of Cp*_3_CoMo_2_S_4_ (red trace, top) and Cp*_3_Mo_3_S_4_ (blue trace, bottom). Redox potentials listed in Table S3. Solvent, DMF; supporting electrolyte, 0.1 M TBAPF_6_; scan rate, 100 mV/s. Potential values are referenced to Fc^+/0^.

Next, we investigated the activity of the assemblies toward proton reduction (Figure 3). Addition of triethylammonium tetrafluoroborate (HNEt_3_BF_4_, p*K*_a_ = 9.3 in DMF^32^) to a DMF solution of Cp*_3_CoMo_2_S_4_ results in a catalytic current with an onset potential at −1.47 V (*E*_pc_ = −1.95 V). The overpotential, calculated at the catalytic current half-peak height (*E*_p/2_), is 470 mV.^33^ We note that the catalytic wave develops at potentials anodically shifted from the [Cp*_3_CoMo_2_S_4_]/[Cp*_3_CoMo_2_S_4_]^−^ redox couple (*E*_1/2_ = −1.80 V). This observation is consistent with either a fast and favorable reaction following reduction of the cluster occurring at a rate significantly higher than the catalytic turnover, or a rate-limiting, concerted proton–electron transfer step.^34,35^ Additional CV experiments in the presence of organic acids with varying p*K*_a_ values show a shift of the catalytic wave to more positive potentials as the p*K*_a_ of the acid decreases, an observation consistent with thermodynamically controlled proton-coupled electron transfer (Figure S2).^36–39^ The catalytic current approaches a plateau under conditions of 200 mM [HNEt_3_BF_4_], 0.5 mM catalyst loading, and a scan rate of 900 mV/s (Figures S3–S4). The catalytic wave observed in the presence of acid corresponds to H_2_ generation, as confirmed by controlled potential electrolysis (CPE at −1.75 V; Figure 3b), with a Faradaic efficiency of 84 ± 6%. A small amount of H_2_ is produced in the absence of catalyst; the total charge passed during the 3 h CPE experiments was 13.7 C for Cp*_3_CoMo_2_S_4_ in comparison to 2.9 C in the absence of catalyst (Table S4).

**Figure 3:**
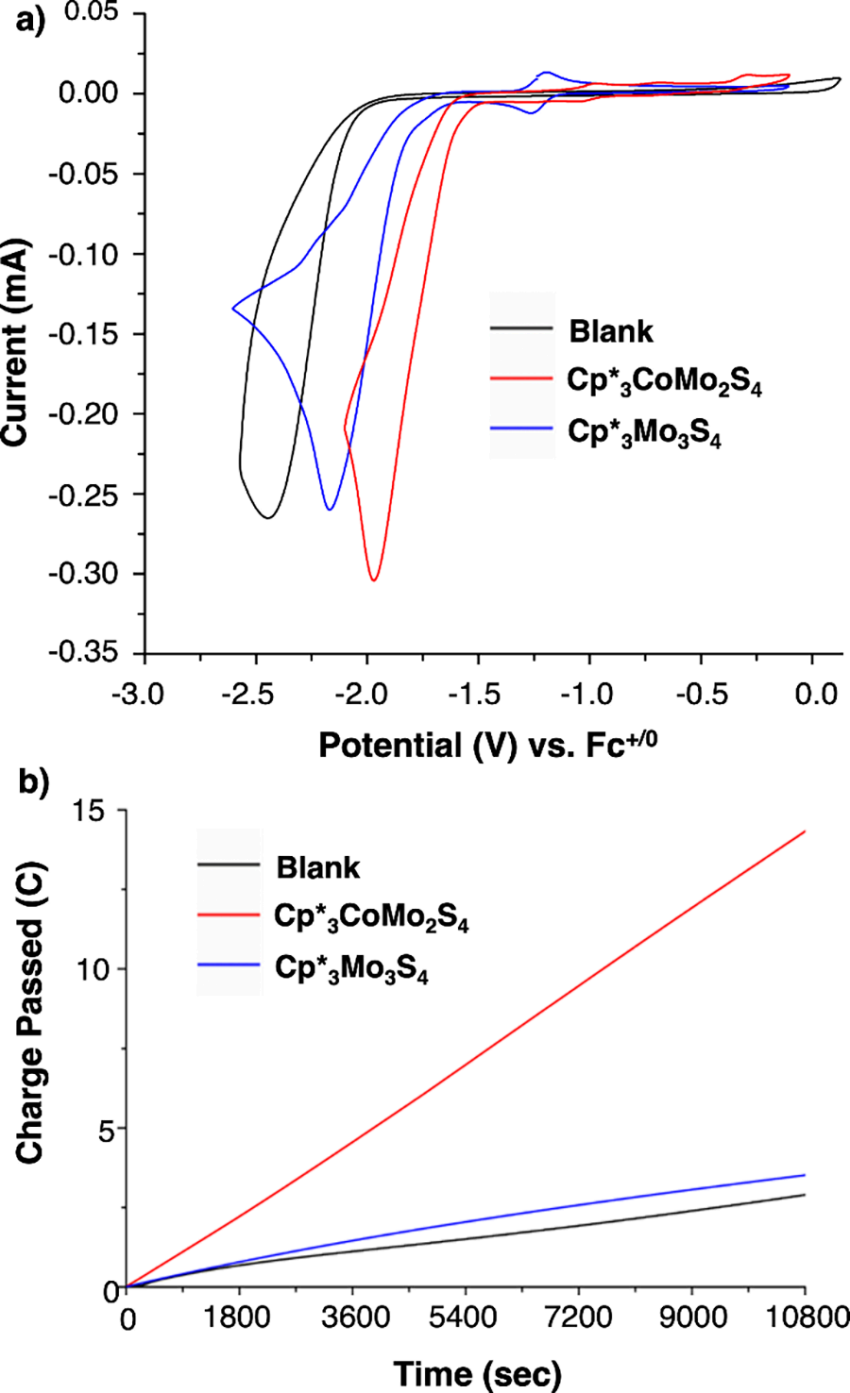
(a) CVs of Cp*_3_CoMo_2_S_4_ (1 mM, red), and Cp*_3_Mo_3_S_4_ (1 mM, blue) in the presence of 32 mM HNEt_3_BF_4_. The black trace corresponds to the control experiment run in the absence of cluster (“blank”). Conditions: 0.1 M TBAPF_6_ in DMF solution, 100 mV/s. (b) Accumulation of charge vs time in the controlled potential electrolysis of a 0.65 mM DMF solution of Cp*_3_MMo_2_S_4_ in the presence of 30 mM of HEt_3_NBF_4_ over 3 h (10800 s) at −1.75 V vs Fc^+/0^ and equivalent electrolysis under the same conditions in the absence of cluster.

The activity of Cp*_3_Mo_3_S_4_ toward proton reduction was assessed under analogous conditions. Addition of HNEt_3_BF_4_ to a DMF solution of Cp*_3_Mo_3_S_4_ results in a catalytic response at *E*_pc_ = −2.14 V vs Fc^+/0^ (onset potential, −1.62 V; Figure 3a), with an overpotential of 660 mV.^33^ H_2_ evolution from Cp*_3_Mo_3_S_4_ was confirmed by CPE; the Faradaic yield of H_2_ formation was 68 ± 8% (Table S4), however the total charge passed over 3 h of electrolysis was only slightly higher than observed in the absence of catalyst (Figure 3b).

To evaluate if proton reduction proceeds via *homogeneous* electrocatalysis, rinse tests were performed with a glassy carbon working electrode in the presence of HEt_3_NBF_4_ and either Cp*_3_CoMo_2_S_4_ or Cp*_3_Mo_3_S_4_. The cyclic voltammograms obtained with rinsed electrodes were similar to those recorded with fresh electrodes under the same conditions without catalyst (Figure S2, see SI for details). Furthermore, no catalyst deposition was observed during bulk electrolysis, as confirmed by rinse tests performed after CPE (Figure S5). Scan-rate dependence studies reveal that the catalytic current is linearly proportional to the square root of the scan rate for both Cp*_3_CoMo_2_S_4_ and Cp*_3_Mo_3_S_4_ (Figure S4). Collectively, these results support that the active catalyst is freely diffusing in solution.

The comparative study on electrocatalytic proton reduction performance by Cp*_3_Mo_3_S_4_ and Cp*_3_CoMo_2_S_4_ is intriguing in light of previous studies reported on MoS_2_ and its Co-doped analogue.^40^ Several research groups have investigated the influence of substitutional Co-doping in MoS_2_, revealing that the Co-dopant improves the performance of the material as an electrocatalyst for HER.^30,41^ Analogously, we have also observed enhanced H_2_ production with Cp*_3_CoMo_2_S_4_ as compared to Cp*_3_Mo_3_S_4_, where the heterometallic assembly reduces the overpotential for proton reduction. These results indicate the trimetallic system serves also as a *functional* model of Co-doped bulk MoS_2_ material.

To gain additional insight into relevant intermediates in proton reduction, stoichiometric reduction and protonation experiments were performed with Cp*_3_CoMo_2_S_4_. As expected, the neutral assembly, Cp*_3_CoMo_2_S_4_, does not react with protons; analysis of the crude reaction mixture following addition of an excess of HNEt_3_BF_4_ to Cp*CoMo_2_S_4_ in THF results in no reaction (Figures S6–S7).

Next, we investigated the isolation and reactivity of the reduced form of Cp*_3_CoMo_2_S_4_. The reduced cluster could be independently synthesized by treating Cp*_3_CoMo_2_S_4_ with KC_8_ in the presence of 18-crown-6 (18-C-6; Scheme 2). Following workup (see SI for details), the product was characterized through ^1^H NMR spectroscopy and combustion analysis (Figure S8). Isolation of [K­(18-C-6)]­[Cp*_3_CoMo_2_S_4_] was confirmed through single crystal X-ray diffraction (Figure 4, Tables S1–S2). Refinement of the data revealed the expected anionic “CoMo_2_S_4_” core, capped by a cationic [K­(18-C-6)]^+^ moiety. The Cp* ligand bound to Co has undergone ring-slippage, now bound to the Co-center through an η_3_ contact (as opposed to η_5_). This observation is distinct for the reduced form of the cobalt-doped derivative of the thiomolybdate cluster. We hypothesize that the observed ring-slippage of the Co-Cp* moiety is a result of the strong interaction of the basic sulfido moieties with the counterion, where coordination mode of the ancillary ligand changes to accommodate coordination of [K­(18-C-6)]. Indeed, the potassium countercation is asymmetrically bound to the trisulfide face of [Cp*_3_CoMo_2_S_4_]^1–^; the K–S contacts between bridging sulfide atoms attached to cobalt (μ_2_-S_Co_) are ∼0.5 Å shorter in length (3.2853(8), 3.1965(8) Å) than the K–S distance to the μ_2_-S^2–^ ligand bridging two molybdenum centers (3.7693(9) Å). This observation supports the hypothesis that the dopant increases the basicity of adjacent sulfur moieties relative to the anionic homometallic assembly. Overall, this bonding analysis suggests that Co-doping of the [Mo_3_S_4_] core induces electronic perturbation on the trisulfide surface, consistent with theoretical reports described in the literature in the context of bulk materials.^40^

**Scheme 2:**
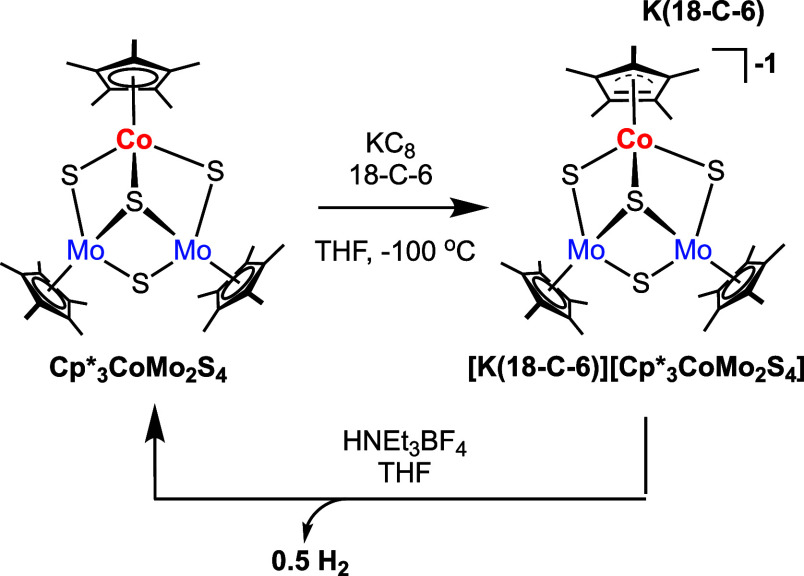
Synthesis of [K­(18-C-6)]­[Cp*_3_CoMo_2_S_4_] and its Subsequent Reactivity with HNEt_3_BF_4_

**Figure 4:**
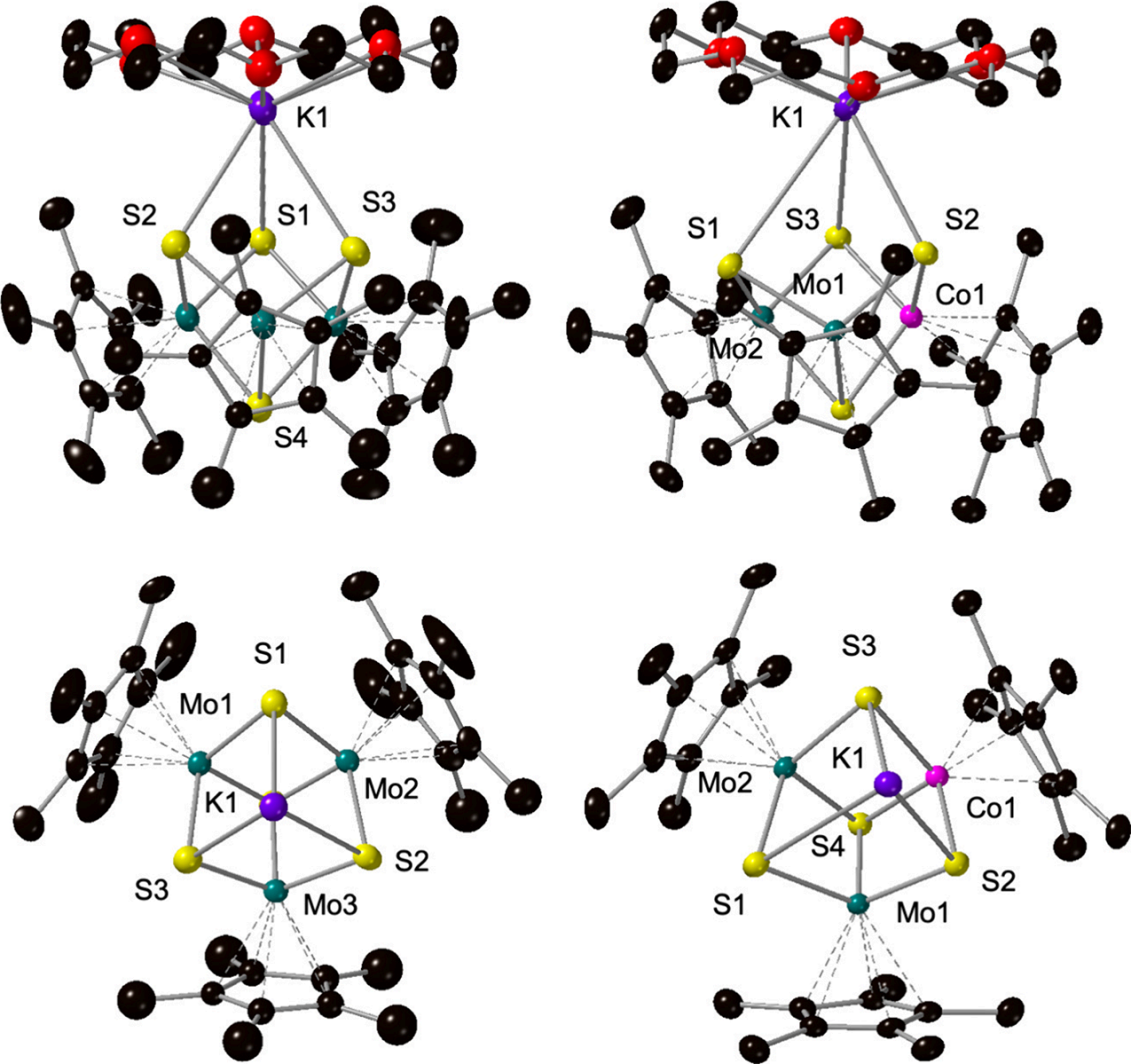
Molecular structures of [K­(18-C-6)]­[(Cp*_3_Mo_3_S_4_)] (left)^27^ and [K­(18-C-6)]­[(Cp*_3_CoMo_2_S_4_)] (right) shown with 50% probability ellipsoids. Solvent molecules and hydrogen atoms have been removed for clarity. Top representations show side-on view of the ion pair. Bottom representations look down at the trisulfide face of the assembly to accentuate discrepancies in potassium coordination; in these images 18-C-6 has been removed for clarity. Key: O, red; C, black; S, yellow; K, purple; Co, pink; Mo, teal.

With the reduced compound, [K­(18-C-6)]­[Cp*_3_CoMo_2_S_4_], in hand, our attention shifted to investigating its reactivity with organic acids. Upon addition of an equivalent HNEt_3_BF_4_ to a THF-*d*_8_ solution of [K­(18-C-6)]­[Cp*_3_CoMo_2_S_4_] in a J-young tube, immediate effervescence was observed, consistent with the release of H_2_(*g*). Analysis of the crude spectrum by ^1^H NMR spectroscopy confirms quantitative conversion of the metal-containing product to its neutral redoxomer, Cp*_3_CoMo_2_S_4_ (Scheme 2, bottom). The oxidation of the cluster was accompanied by the formation of ∼0.5 equiv of H_2_ (Figure S8). Based on this information, we propose the mono reduced Co-doped thiomolybdate cluster is the form of the assembly active for proton reduction.

Molecular thiomolybdate clusters have been reported as electrocatalysts for proton reduction in organic solvent.^42^ Perhaps most relevant to this work, Dubois and co-workers describe a molybdenum sulfide dimer, (CpMo­(μ-S)_2_)_2_(S_2_CH_2_), that serves as a electrocatalyst for HER in acetonitrile with modest overpotentials (∼120 mV).^43^ Mechanistic studies suggest that the first step involves protonation of the S-center of the dimer. This is supported by the observation that electrocatalytic proton reduction occurs in acetonitrile and dichlorobenzene, whereas strongly basic solvents such as DMF inhibit catalysis. In contrast, our hemicuboidal structure shows good activity in basic solvent, suggesting that the first step is unlikely to involve direct protonation of the S-center; instead, a hydrosulfido intermediate is generated after proton-coupled reduction of the assembly. These disparate mechanistic details may be a result of the fact that these thiomolybdate assemblies differ in sulfur to metal ratios and thus model distinct facets of the layered material; the introduction of heterometal center also leads to the differed HER mechanism coupled with the metal reduction. Indeed, the dimeric thiomolybdate clusters popularized by Dubois are touted as models for the active *edge* sites of MoS_2_.^42^ Ongoing studies are focused on elucidation of additional mechanistic details related to electrocatalytic proton reduction.

In summary, the synthesis of a cobalt-substituted thiomolybdate cluster is presented as a structural and functional model of basal plane of Co-doped MoS_2_. Cp*_3_CoMo_2_S_4_ functions as a homogeneous electrocatalyst for HER; a catalytic current in the CVs is observed on the addition of excess HNEt_3_BF_4_ to Cp*_3_CoMo_2_S_4_, along with CPE experiments confirming the production of H_2_ with high Faradaic efficiencies. Comparative studies with the homometallic analogue, Cp*_3_Mo_3_S_4_ reveal that the Co-doped cluster exhibits improved catalytic performance toward proton reduction. The reduced form of Cp*_3_CoMo_2_S_4_ was isolated, and its subsequent reactivity was studied to provide insight into the reaction pathway. These results provide support for the experimental observations indicating that cobalt substitution activates the basal plane of the MoS_2_ material for HER. Overall, this study constitutes our first foray into the synthesis and reactivity of transition metal doped thiomolybdate clusters that model the basal plane of MoS_2_.

## Supplementary Material



## Notes

Experimental information, ^1^H NMR spectra, additional CV data (PDF)
