# Supplementary material for: Proton Reduction with a Cobalt-Doped Thiomolybdate Cluster: A Structural and Functional Model of Co-Doped MoS2
Source: J Am Chem Soc. 2025 Oct 15;147(43):38973–8. doi: 10.1021/jacs.5c09888 (PMC12576832; doi:10.1021/jacs.5c09888)
Supplement: Supplementary file 1 [file ja5c09888_si_001.pdf]

Electronic Supporting Information

Proton Reduction with a Cobalt-doped Thiomolybdate Cluster: A Structural and Functional Model of Co-doped MoS_2

Kamaless Patra, Leyla R. Valerio‡, Zhou Lu‡, Kaye Kuphal, William W. Brennessel, and Ellen M. Matson*

Department of Chemistry, University of Rochester, Rochester NY 14627 USA

‡ These authors contributed equally.

Corresponding Author Contact Information:

Ellen M. Matson: matson@chem.rochester.edu

Table of Contents

Experimental Section S3

Figure S1. ^{1}H NMR spectra of $Cp^*_2Mo_2S_4$ (top, black), $(Cp^*CoCl)_2$ (middle, grey), and **$Cp^*_3CoMo_2S_4$** (bottom, red) obtained in C_6D_6 at room temperature (~22 °C). S6

Table S1. Single crystal X-ray diffraction data for **$Cp^*_3CoMo_2S_4$** and **[K(18-C-6)][$Cp^*_3CoMo_2S_4$]**. S7

Table S2. Selected bond lengths (Å) of **$Cp^*_3Mo_3S_4$**,[8] **[K(18-C-6)][($Cp^*_3Mo_3S_4$)]**,[3] **$Cp^*_3CoMo_2S_4$**, and **[K(18-C-6)][$Cp^*_3CoMo_2S_4$].** S8

Table S3. Redox potentials of **$Cp^*_3CoMo_2S_4$** and **$Cp^*_3Mo_3S_4$** (V vs. $Fc^{+/0}$) determined from the CVs of the two assemblies collected in DMF (100 mV sec^{-1} at a GC electrode, with 0.1 M $TBAPF_6$ as the supporting electrolyte. S8

Table S4. Controlled potential electrolysis (CPE) for H_2 generation, summary of CPE experiments. S9

Figure S2. Cyclic voltammograms of $Cp^*_3CoMo_2S_4$ in DMF (0.1 M $TBAPF_6$, 100 mV/s) with 30 mM CH_3COOH (pK_a = 13.5)[9] (pink), $HNEt_3BF_4$ (pK_a = 9.25) (red), HBF_4 (pK_a = 3.4)[9] (blue), $HOCHNMe_2OTf$ (pK_a = 1.6)[10] (black). S9

Figure S3. Cyclic voltammograms of 0.5 mM a) $Cp^*_3CoMo_2S_4$ and b) $Cp^*_3Mo_3S_4$ in presence of 30 mM $HNEt_3BF_4$ in DMF (0.1 M $TBAPF_6$) recorded at scan rates ranging from 0.1 to 0.9 V/s c) Plot of Ic (mA) vs $[\text{Scan rate}]^{1/2}$ $(V/sec)^{1/2}$. S10

Figure S4. a) Cyclic voltammograms of 0.5 mM **$Cp^*_3CoMo_2S_4$** and 200 mM of $HNEt_3BF_4$ in DMF (0.1 M $TBAPF_6$) recorded at scan rates ranging from 0.1 to 0.9 V/s b) Plot of I_c (mA) vs $[\mathbf{HNEt_3BF_4}]^{1/2}$ $(mM)^{1/2}$, for [**$HNEt_3BF_4$**] concentrations ranging from 64 to 200 mM, recorded at a scan rate of 0.9 V/s, where the catalytic current approaches a plateau. S10

Figure S5. a) Cyclic voltammograms using a glassy carbon (GC) electrode (3 mm diameter) as the working electrode with 10 mM HEt_3NBF_4 as the proton source in the absence of catalyst (black trace), in the presence of $Cp^*_3Mo_2CoS_4$ (1 mM) (red trace), and after rinsing the GC electrode (blue trace) in a separate solution containing 10 mM acid but no catalyst; b) current–time traces from 10-min bulk electrolysis using a GC rod (area = 2 cm^2) as the working electrode with 10 mM HEt_3NBF_4 in the absence of catalyst (black trace), in the presence of $Cp^*_3Mo_2CoS_4$ (red trace), and using the rinsed electrode in a fresh solution containing 10 mM HEt_3NBF_4 without catalyst (blue trace). c) Rinse experiment using cyclic voltammetry as in panel (a), repeated with $Cp^*_3Mo_3S_4$ catalyst. d) Rinse experiments using CPE as in panel (c), performed with $Cp^*_3Mo_3S_4$ as catalyst. S11

Figure S6. ^{1}H NMR spectrum (500 MHz) of **$Cp^*_3CoMo_2S_4$** and excess $HNEt_3BF_4$ measured in THF-d_8 at room temperature. Partial dissolution of the excess $HNEt_3BF_4$ in THF-d_8 was observed. The **$Cp^*_3CoMo_2S_4$** displays a Cp*-methyl resonance at 1.47 ppm. To assess whether this shift arises from a reaction between the cluster and the acid, cyclic voltammetry was performed in the presence of excess $HNEt_3BF_4$ (Figure S7). S12

Figure S7. Cyclic voltammograms of (a) **$Cp^*_3CoMo_2S_4$** (black trace, top) and b) after addition of 15 equivalent of $HNEt_3BF_4$ (red trace, bottom). Solvent, THF; supporting electrolyte, 0.1 M $TBAPF_6$; scan rate, 100 mV/s. Potential values are expressed with reference to $Fc^{+/0}$. S12

Figure S8. ^{1}H NMR spectra of $Cp^*_3CoMo_2S_4$ (top, red) and **[K(18-c-6)][($Cp^*_3CoMo_2S_4$)]** (bottom, pink) obtained in THF-d_8 at room temperature (~22 °C). S13

Figure S9. ^{1}H NMR spectrum (500 MHz) of **[K(18-c-6)][($Cp^*_3CoMo_2S_4$)]** and one equivalent of $HNEt_3BF_4$ measured in THF-d_8 at room temperature. [The signals at 4.55 and 1.46 ppm correspond to H_2, and the **$Cp^*_3CoMo_2S_4$** cluster, respectively; Signals at 2.68 and 1.08 ppm are assigned to triethylamine, while peaks at 1.77 and 3.62 ppm arise from residual tetrahydrofuran] S13

References: S14

Experimental Section

General Considerations. All air- and moisture-sensitive manipulations were carried out using standard high vacuum line, Schlenk, or cannula techniques, or in an MBraun inert atmosphere glovebox containing an atmosphere of purified dinitrogen. Solvents for air- and moisture-sensitive manipulations were dried and deoxygenated using a Glass Contour Solvent Purification System (Pure Process Technology, LLC) and stored over activated 4 Å molecular sieves (Fisher Scientific) prior to use. Deuterated solvents for ^{1}H NMR spectroscopy were purchased from Cambridge Isotope Laboratories and stored in the glovebox over activated 3 Å molecular sieves after three freeze-pump-thaw cycles. $(Cp^*CoCl)_2$,[1] KC_8,[2] $[(Cp^*_3Mo_3S_4)K(18\text{-}C\text{-}6)]$,[3] $(Cp^*_3Mo_3S_4)$,[3] $Cp^*_2Mo_2S$,[4] and Et_3NHBF_4[5] were synthesized following literature procedures.

Physical Measurements. ^{1}H NMR spectra were recorded at room temperature on a 400 MHz Bruker AVANCE spectrometer or a 500 MHz Bruker AVANCE spectrometer locked on the signal of deuterated solvents. All chemical shifts are reported relative to the chosen deuterated solvent as a standard. Cyclic voltammetry (CV) was performed using a three-electrode setup inside a nitrogen filled glove box (MBraun UniLab, USA) using a Bio-Logic SP 150 potentiostat/galvanostat and the EC-Lab software suite. The concentration of the cluster and the supporting electrolyte ($TBAPF_6$) were kept at 1 mM and 100 mM respectively throughout all measurements. CVs were recorded using a 3 mm diameter glassy carbon working electrode (CH Instruments, USA), a Pt wire auxiliary electrode (CH Instruments, USA), and an Ag/Ag^+ non-aqueous reference electrode with 0.01 M $AgNO_3$ in 0.1 M $TBAPF_6$ in the desired solvents used for the experiment (DMF and THF) (BASi, USA). CVs were iR compensated at 85% with impedance taken at 100 kHz using the ZIR tool included within the EC-Lab software. Bulk electrolysis experiments were performed in a three-neck round bottom glass-cell using a Bio-Logic SP 150 potentiostat/galvanostat. In all experiments, 0.1 M supporting electrolyte ($TBAPF_6$) in 5 ml of desired solvent (DMF or THF) was used. Elemental analysis data was obtained from the Elemental Analysis Facility at the University of Rochester. Microanalysis samples were weighed with a PerkinElmer model AD6000 autobalance, and their compositions were determined with a PerkinElmer 2400 series II analyzer. Air-sensitive samples were handled in a VAC Atmospheres glovebox.

Single Crystal X-ray Crystallography. Single crystals of $[Cp^*_3Mo_2CoS_4]$ and $[(Cp^*_3Mo_2CoS_4)K(18\text{-}C\text{-}6)]$ were placed on a nylon loop and mounted on a Rigaku XtaLAB Synergy-S Dualflex diffractometer equipped with a HyPix-6000HE HPC area detector for data collection at 100.00(10) K. A preliminary set of cell constants and an orientation matrix were calculated from a small sampling of reflections. A short pre-experiment was run, from which an optimal data collection strategy was determined. The full data collection for all four complexes was carried out using a PhotonJet (Cu) X-ray source. After the intensity data were corrected for absorption, the final cell constants were calculated from the xyz centroids of the strong reflections from the actual data collections after integration. The structure was solved using SHELXT[6] and refined using SHELXL[7]. Most or all non-hydrogen atoms were assigned from the solution. Full-

matrix least squares/difference Fourier cycles were performed, which located any remaining non-hydrogen atoms. All the non-hydrogen atoms were refined with anisotropic displacement parameters. All the hydrogen atoms were placed in ideal positions and refined as riding atoms with relative isotropic displacement parameters.

Controlled-potential Electrolysis and Gas Chromatography

Controlled potential electrolysis experiments were performed in a three-neck glass cell, using a three-electrode system: a Ag/$AgNO_3$ reference electrode (BASi, USA), a glassy cardon rod working electrode with a surface area of approximately 2 cm^2 (Cylindrical GC rod of thickness 0.3 mm and length immersed in solution is 2 cm), and a Pt-wire counter electrode. All three electrodes were placed in the same compartment, inserted through rubber septa fitted into the three ports of the glass cell. All substrates, cluster, and respective solvents were added inside the glovebox under an inert atmosphere. The vessel was then sealed with electrical tape to prevent contamination from the external atmosphere. The volume of the solution in the compartment was 7.5 mL, purged with an 80:20% N_2:CH_4 mixture (from Airgas) before each experiment, where CH_4 serves as an internal standard. The amount of H_2 generated during the experiment was determined by gas chromatography (GC) using a calibration curve obtained by injecting known volumes of H_2 at 1 atm. The GC instrument is a Shimadzu GC-2014 with a Thermal Conductivity Detector and a Restek RT-Msieve 5 Å column.

Turn Over Number (TON) Calculation

$$TON = \frac{n_P(X) - n_{P,b}(X)}{n_T(cat)}$$

$n_p(X)$ = moles of H_2 detected by GC

$n_{p,b}(X)$ = moles of product detected by GC after background bulk electrolysis in the absence of catalyst is subtracted.

$n_T(cat)$: total moles of catalyst in the electrolyte

Faradaic Efficiency (FE%) Calculation

$$FE\% = \frac{n_P(X)Fv}{Q_T} \times 100\%$$

$$FE^{corr}\% = \frac{\left[n_P(X) - n_{P,b}(X)\right]Fv}{Q_T - Q_b} \times 100\%$$

$n_p(X)$ = moles of H_2 detected by GC

$n_{p,b}(X)$ = moles of product detected by GC after background bulk electrolysis in the absence of catalyst is subtracted.

F = Faraday’s constant (96,485 C/mol)

v =number of electrons transferred per molecule of product (2 e^-)

Q_T = total charge passed in the CPE experiment

Q_b = total charge passed in the background CPE experiment in the absence of catalyst

Synthesis of $Cp^*_3CoMo_2S_4$

In a glovebox, a 20 mL scintillation vial equipped with a magnetic stir bar was charged with **$Cp^*_2Mo_2S_4$** (0.050 g, 0.085 mmol) and 8 mL of tetrahydrofuran (THF). The mixture was stirred vigorously until complete dissolution of the solid, and the resulting solution was then frozen completely inside the cold well. Sodium naphthalenide (0.4 M in THF, 0.21 mL, 0.605 mmol) was added while the frozen solution was allowed to thaw under vigorous stirring, and the mixture was gradually warmed to room temperature. The solution turned red-brown and was subsequently frozen again. Then, $(Cp^*CoCl)_2$ (19.5 mg, 0.042 mmol, 0.5 equivalent) was added while the solution thawed, and the mixture was gradually warmed to room temperature. The volatiles were removed under vacuum immediately, affording a dark brown solid. The residue was dissolved in toluene, filtered through Celite, and evaporated to dryness. The resulting brown crystalline solid was washed twice with 2 mL of acetonitrile to afford the title compound as a brown crystalline solid. Yield: 0.055 g, 0.070 mmol, 82%. ^{1}H NMR (400 MHz, C_6D_6) δ = 1.39 (45, Cp*-*H*); **Figure S1.** Brown crystals suitable for single crystal X-ray diffraction were grown from a concentrated toluene solution of the product at -30 ^{o}C. Anal. Calcd. for $C_{40}H_{60}Mo_2CoS_4$•0.5(C_7H_8) (mol. Wt. 1377.23 g mol^{-1}): C, 48.43; H, 5.94. Found: C, 48.45; H, 5.80.

Synthesis of [K(18-C-6)][$Cp^*_3CoMo_2S_4$]

In the glovebox, a 20 mL scintillation vial equipped with a magnetic stir bar was charged with **$Cp^*_3Mo_2CoS_4$** (0.030 g, 0.038 mmol), 18-crown-6 (18-C-6, 0.010 g, 0.038 mmol) and 8 mL of tetrahydrofuran. The solution was stirred vigorously for 5 minutes to dissolve all solids and then frozen completely inside the cold well. KC_8 (0.005 mg, 0.038 mmol) was added as solid while the solution thawed. The resulting mixture was gradually warmed to room temperature, during which the color changed to dark brown. The mixture was immediately filtered through Celite to obtain a dark brown solution, which was concentrated to ~2 mL and cooled to −30 °C for 24 h to afford dark plates. The crystals were washed twice with 5 mL of pentane to yield **[K(18-C-6)][$Cp^*_3CoMo_2S_4$]** as a dark brown solid. Yield: 0.026 g, 0.024 mmol, 65%. **[K(18-C-6)][$Cp^*_3CoMo_2S_4$]** is extremely air-sensitive, as evidenced by a rapid color change from dark brown to green-brown upon exposure to air. The reduced species is also thermally sensitive; storing the anionic complex at room temperature for more than 48 hours under inert conditions leads to the disappearance of the Cp* signal. ^{1}H NMR (400 MHz, THF-*d_8*) δ = 4.80 (150, 30H, Cp*-*H*), 3.81 (33, 24H, 18-C-6). Brown crystals suitable for single crystal X-ray diffraction were grown from a concentrated toluene solution of the product at −30 °C. Anal. Calcd. for $C_{42}H_{69}Mo_2S_4CoO_6K$ (mol. Wt. 1088.18 g mol^{-1}): C, 46.36; H, 6.39. Found: C, 46.28; H, 6.63.

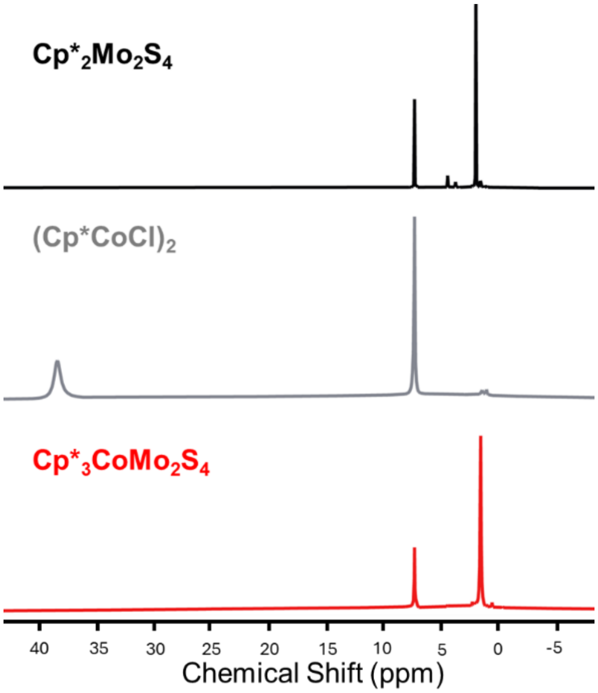

Figure S1. ^{1}H NMR spectra of $Cp^*_2Mo_2S_4$ (top, black), $(Cp^*CoCl)_2$ (middle, grey), and $\mathbf{Cp^*_3CoMo_2S_4}$ (bottom, red) obtained in C_6D_6 at room temperature (~22 °C).

Table S1. Single crystal X-ray diffraction parameters for **$Cp^*_3CoMo_2S_4$** and **$[K(18\text{-}C\text{-}6)][Cp^*_3CoMo_2S_4]$**.

	**$Cp^*_3CoMo_2S_4$**	**$[K(18\text{-}C\text{-}6)][Cp^*_3CoMo_2S_4]$**
Empirical formula	$C_{30}H_{45}CoMo_2S_4$	$C_{50}H_{85}CoKMo_2O_8S_4$
Formula weight	784.71	1232.32
Temperature	100.00(10) K	100.00(10) K
Wavelength	1.54184 Å	1.54184 Å
Crystal system	Monoclinic	Triclinic
Space group	$P2_{1/n}$	P-1
Unit cell dimensions	a = 10.88580(10) Å<br>b = 16.88150(10) Å<br>c = 19.84610(10) Å<br>α = 90°<br>β = 96.3090(10) °<br>γ = 90°	a = 12.1116(2) Å<br>b = 13.8120(2) Å<br>c = 17.3750(3) Å<br>α = 76.9450(1)°<br>β = 82.7410(10)°<br>γ = 83.939(2)°
Volume	3625.00(4) $Å^3$	2799.83(8) $Å^3$
Z	4	2
Independent reflections	7805	12017
Goodness-of-fit on F^2	1.053	1.044
Final R indices [I>2sigma(I)]	R_1 = 0.0309<br>wR_2 = 0.0778	R_1 = 0.0303<br>wR_2 = 0.0755

Table S2. Selected bond lengths (Å) of **$Cp^*{}_3Mo_3S_4$**,[8] **[K(18-C-6)][($Cp^*{}_3Mo_3S_4$)]**,[3] **$Cp^*{}_3CoMo_2S_4$**, and **[K(18-C-6)][$Cp^*{}_3CoMo_2S_4$]**.

Bond	$Cp^*{}_3Mo_3S_4$	[K(18-C-6)][($Cp^*{}_3Mo_3S_4$)]	$Cp^*{}_3CoMo_2S_4$	[K(18-C-6)][$Cp^*{}_3CoMo_2S_4$]
Co-Mo	--	--	2.8268(4)<br>2.8306(4)	2.7362(4)<br>2.7539(4)
Co-(m_2-S_{Mo})	--	--	2.2927(7)<br>2.2997(8)	2.2470(7)<br>2.2639(7)
Co-(m_3-S)	--	--	2.2772(7)	2.1933(6)
Co-C(Cp*)	--	--	2.328(3) (avg)	2.116(2), 2.521(2),<br>2.600(2), 3.164(3), 3.121(3)<br>**h_3 binding of Cp* ligand*
Mo-Mo	2.8651(7)<br>2.8544(7)<br>2.8637(7)	2.8530(4)<br>2.8555(4)<br>2.8574(4)	2.8339(3)	2.8032(2)
Mo-(m_3-S_{Mo})	2.341(1)<br>2.337(1)<br>2.337(1)	2.3419(9)<br>2.3437(9)<br>2.3444(8)	2.2999(6)<br>2.3055(6)	2.3159(6)<br>2.3170(6)
Mo-(m_2-S_{Co})	--	--	2.2064(6)<br>2.2221(7)	2.2432(6)<br>2.2495(6)
Mo-(m_2-S_{Mo})	2.2957(14) -<br>2.3139(15)	2.3393(9) -<br>2.3463(10)	2.3226(6)<br>2.3304(6)	2.3497(6)<br>2.3463(5)
Mo-C(Cp*)	2.370(4) (avg)	2.374 (avg)<br>**disordered*	2.362(5) (avg)	2.387(4) (avg)
K-S	--	3.2612(13)<br>3.2746(13)<br>3.4116(11)	--	3.7693(9)<br>3.2852(8)<br>3.1965(8)

Table S3. Redox potentials of **$Cp^*{}_3CoMo_2S_4$** and **$Cp^*{}_3Mo_3S_4$** (V vs. $Fc^{+/0}$) determined from the CVs of the two assemblies collected in DMF (100 mV sec^{-1} at a GC electrode, with 0.1 M $TBAPF_6$ as the supporting electrolyte.

Redox Couple	$Cp^*{}_3CoMo_2S_4$	$Cp^*{}_3Mo_3S_4$
$[Cp^*{}_3M'Mo_2S_4]^{+/+2}$	−0.252	--
$[Cp^*{}_3M'Mo_2S_4]^{0/+}$	−0.96	−1.23
$[Cp^*{}_3M'Mo_2S_4]^{0/-}$	−1.80	−2.22

Table S4. Controlled potential electrolysis (CPE) for H_2 generation, summary of CPE experiments.

Catalyst	Total Charge (C)	Duration (s)	$E_{applied}$ (V)	FE(H_2) %	FE^{corr}(H_2) %	TON
**$Cp^*_3CoMo_2S_4$**	13.2 ± 0.5	10800	-1.75	98 ± 6	84 ± 6	10
**$Cp^*_3Mo_3S_4$**	3.3 ± 0.3	10800	-1.75	82 ± 8	68 ± 8	2.1
No catalyst (blank)	2.9	10800	-1.75	14	--	--

Proton reduction experiments with catalyst were run in triplicate to obtain error measurements for catalyst activity. Control experiments in the absence of catalyst were run once; as such error values are not reported.

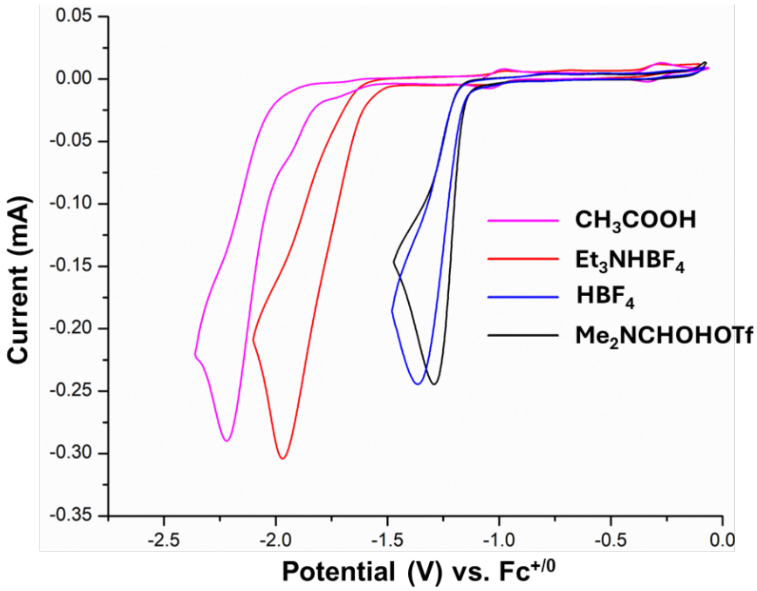

Figure S2. Cyclic voltammograms of $Cp^*_3CoMo_2S_4$ in DMF (0.1 M $TBAPF_6$, 100 mV/s) with 30 mM CH_3COOH (pK_a = 13.5)[9] (pink), $HNEt_3BF_4$ (pK_a = 9.25) (red), HBF_4 (pK_a = 3.4)[9] (blue), $HOCHNMe_2OTf$ (pK_a = 1.6)[10] (black).

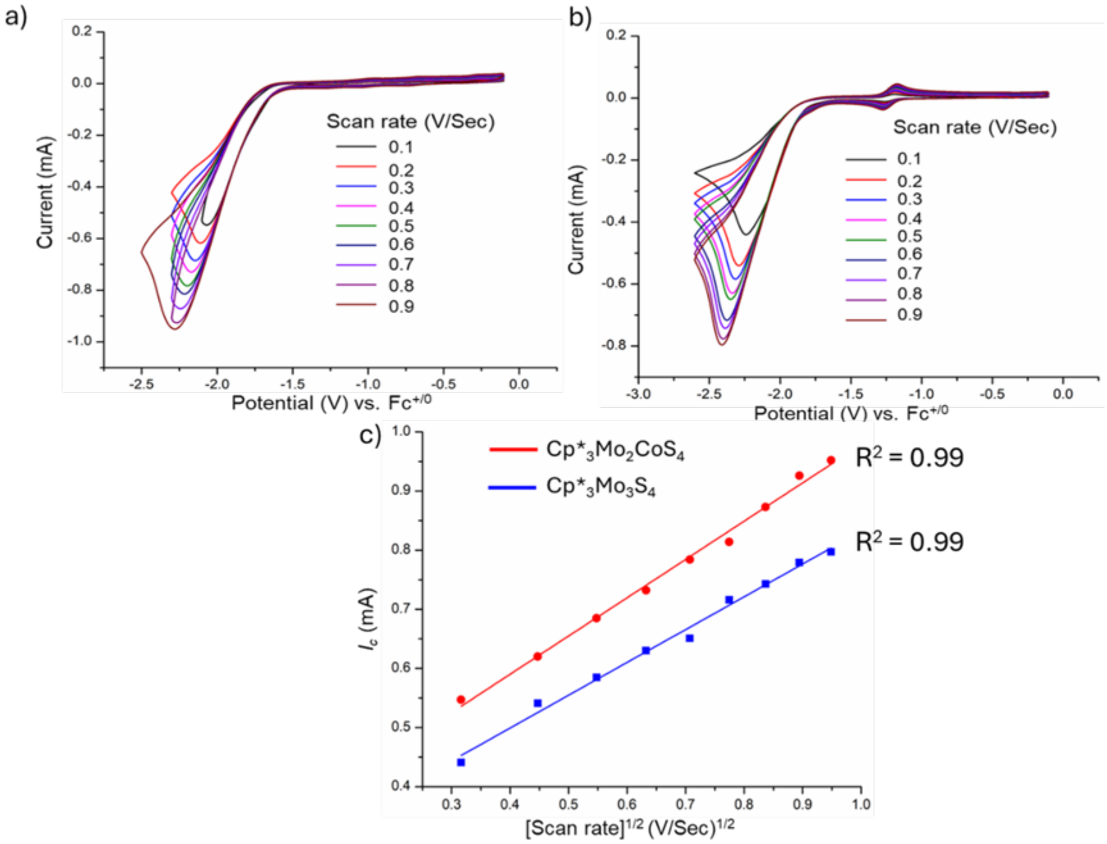

Figure S3. Cyclic voltammograms of 0.5 mM a) $Cp^*_3CoMo_2S_4$ and b) $Cp^*_3Mo_3S_4$ in presence of 30 mM $HNEt_3BF_4$ in DMF (0.1 M $TBAPF_6$) recorded at scan rates ranging from 0.1 to 0.9 V/s c) Plot of Ic (mA) vs [Scan rate]$^{1/2}$ (V/sec)$^{1/2}$.

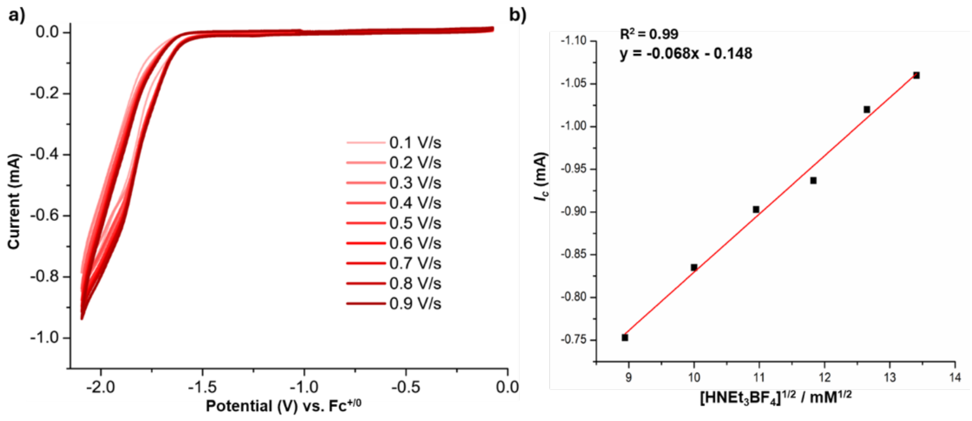

Figure S4. a) Cyclic voltammograms of 0.5 mM $\mathbf{Cp^*_3CoMo_2S_4}$ and 200 mM of $HNEt_3BF_4$ in DMF (0.1 M $TBAPF_6$) recorded at scan rates ranging from 0.1 to 0.9 V/s b) Plot of I_c (mA) vs [$\mathbf{HNEt_3BF_4}$]$^{1/2}$ (mM)$^{1/2}$, for [$\mathbf{HNEt_3BF_4}$] concentrations ranging from 64 to 200 mM, recorded at a scan rate of 0.9 V/s, where the catalytic current approaches a plateau.

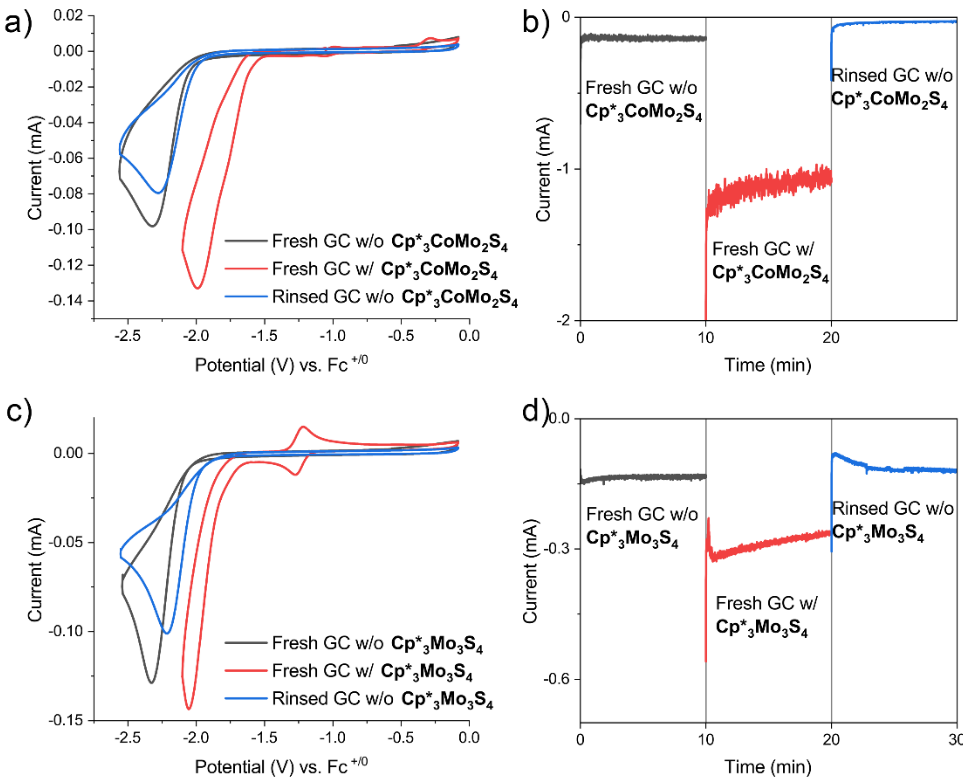

Figure S5. a) Cyclic voltammograms using a glassy carbon (GC) electrode (3 mm diameter) as the working electrode with 10 mM HEt_3NBF_4 as the proton source in the absence of catalyst (black trace), in the presence of $Cp^*_3Mo_2CoS_4$ (1 mM) (red trace), and after rinsing the GC electrode (blue trace) in a separate solution containing 10 mM acid but no catalyst; b) current–time traces from 10-min bulk electrolysis using a GC rod (area = 2 cm²) as the working electrode with 10 mM HEt_3NBF_4 in the absence of catalyst (black trace), in the presence of $Cp^*_3Mo_2CoS_4$ (red trace), and using the rinsed electrode in a fresh solution containing 10 mM HEt_3NBF_4 without catalyst (blue trace). c) Rinse experiment using cyclic voltammetry as in panel (a), repeated with $Cp^*_3Mo_3S_4$ catalyst. d) Rinse experiments using CPE as in panel (c), performed with $Cp^*_3Mo_3S_4$ as catalyst.

All the voltammograms are recorded in DMF solvent containing 0.1 (M) TBAPF6 at 0.1 V/s scan rate. Current vs. time plots of bulk electrolysis experiments were performed using GC rod (area = 2 cm^2), 0.1 M supporting electrolyte ($TBAPF_6$) in 5 mL of DMF).

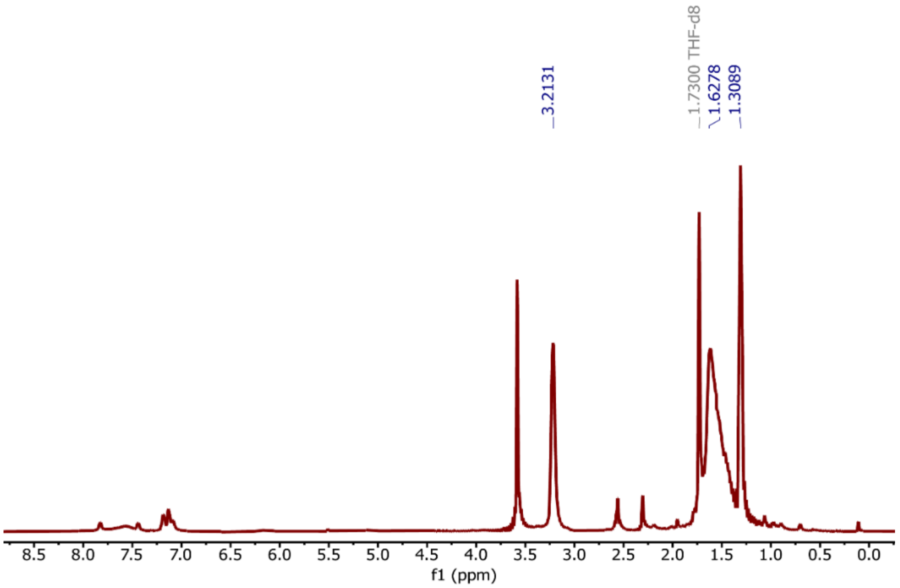

Figure S6. ^{1}H NMR spectrum (500 MHz) of **$Cp^*_3CoMo_2S_4$** and excess $HNEt_3BF_4$ measured in THF-d_8 at room temperature. Partial dissolution of the excess $HNEt_3BF_4$ in THF-d_8 was observed. The **$Cp^*_3CoMo_2S_4$** displays a Cp*-methyl resonance at 1.47 ppm. To assess whether this shift arises from a reaction between the cluster and the acid, cyclic voltammetry was performed in the presence of excess $HNEt_3BF_4$ (Figure S7).

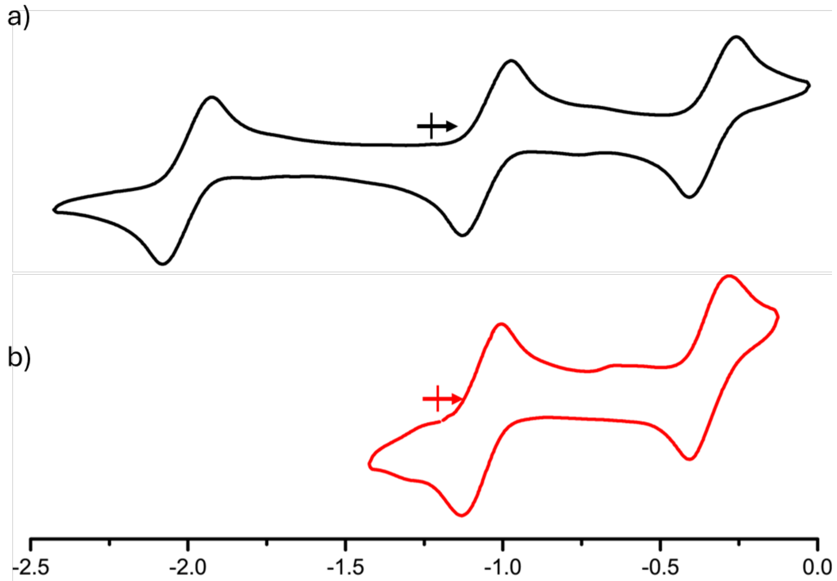

Figure S7. Cyclic voltammograms of (a) **$Cp^*_3CoMo_2S_4$** (black trace, top) and b) after addition of 15 equivalent of $HNEt_3BF_4$ (red trace, bottom). Solvent, THF; supporting electrolyte, 0.1 M $TBAPF_6$; scan rate, 100 mV/s. Potential values are expressed with reference to $Fc^{+/0}$.

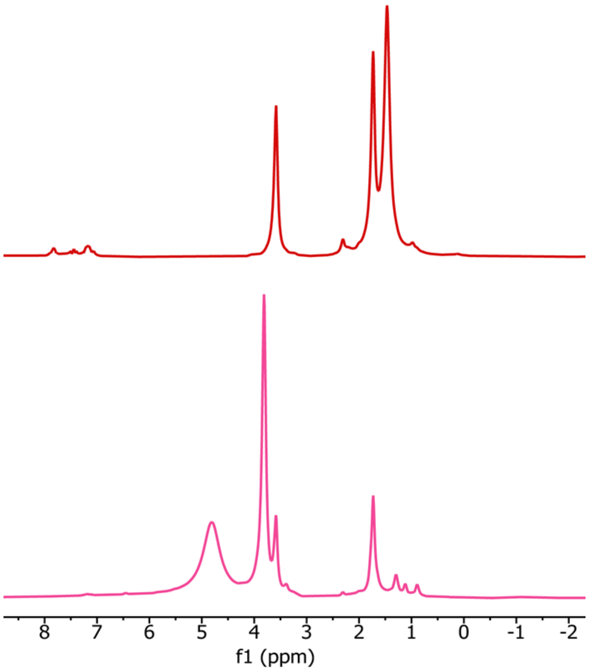

Figure S8. ^{1}H NMR spectra of $Cp^*_3CoMo_2S_4$ (top, red) and **$[K(18\text{-}c\text{-}6)][(Cp^*_3CoMo_2S_4)]$** (bottom, pink) obtained in THF-d_8 at room temperature (~22 °C).

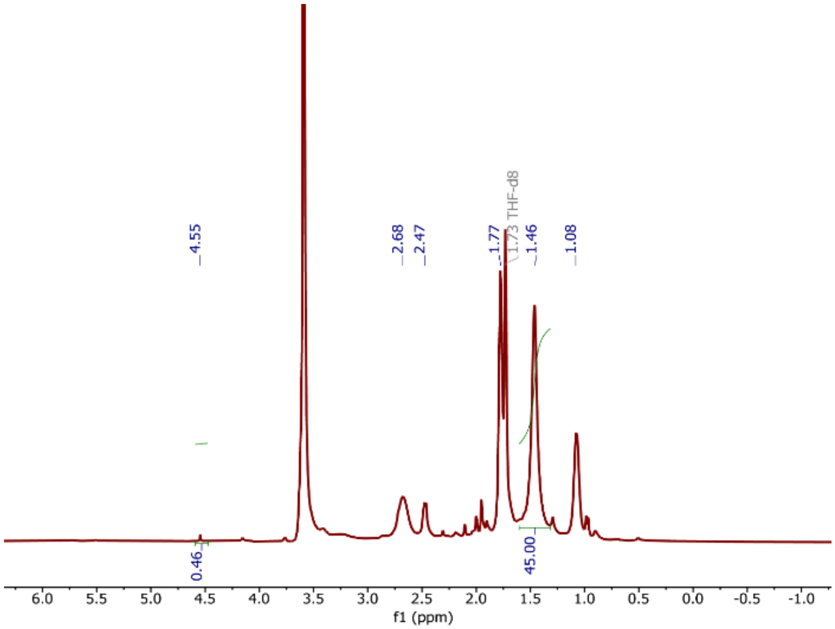

Figure S9. ^{1}H NMR spectrum (500 MHz) of **$[K(18\text{-}c\text{-}6)][(Cp^*_3CoMo_2S_4)]$** and one equivalent of $HNEt_3BF_4$ measured in THF-d_8 at room temperature. [The signals at 4.55 and 1.46 ppm correspond to H_2, and the **$Cp^*_3CoMo_2S_4$** cluster, respectively; Signals at 2.68 and 1.08 ppm are assigned to triethylamine, while peaks at 1.77 and 3.62 ppm arise from residual tetrahydrofuran].

References:

(1) Koelle, U.; Fuss, B.; Belting, M.; Raabe, E. Pentamethylcyclopentadienyl transition metal complexes. 9. Reactions and solid-state and solution behavior of dinuclear cobalt(II) complexes $[C_5Me_5Co(\mu\text{-}X)]_2$. *Organometallics* **1986**, *5*, 980-987.

(2) Chakraborty, S.; Chattopadhyay, J.; Guo, W.; Billups, W. E. Functionalization of Potassium Graphite. *Angew. Chem. Int. Ed.* **2007**, *46*, 4486-4488.

(3) Ohki, Y.; Uchida, K.; Hara, R.; Kachi, M.; Fujisawa, M.; Tada, M.; Sakai, Y.; Sameera, W. M. C. Cubane-Type $[Mo_3S_4M]$ Clusters with First-Row Groups 4–10 Transition-Metal Halides Supported by C_5Me_5 Ligands on Molybdenum. *Chem. – Eur. J.* **2018**, *24*, 17138-17147.

(4) Rakowski DuBois, M.; DuBois, D. L.; VanDerveer, M. C.; Haltiwanger, R. C. Syntheses, structures, and reactions of molybdenum complexes with sulfido and disulfido ligands. *Inorg. Chem.* **1981**, *20*, 3064-3071.

(5) Schreiber, E.; Petel, B. E.; Matson, E. M. Acid-Induced, Oxygen-Atom Defect Formation in Reduced Polyoxovanadate-Alkoxide Clusters. *J. Am. Chem. Soc.* **2020**, *142*, 9915-9919..

(6) Sheldrick, G. SHELXT - Integrated space-group and crystal-structure determination. *Acta Crystallogr. Sec. A* **2015**, *71*, 3-8.

(7) Sheldrick, G. New features added to the refinement program SHELXL since 2008 are described and explained. *Acta Crystallogr. C* **2015**, *71*, 3-8.

(8) Cramer, R. E.; Yamada, K.; Kawaguchi, H.; Tatsumi, K. Synthesis and Structure of a Mo_3S_4 Cluster Complex with Seven Cluster Electrons. *Inorg. Chem.* **1996**, *35*, 1743-1746.

(9) Queyriaux, N.; Sun, D.; Fize, J.; Pécaut, J.; Field, M. J.; Chavarot-Kerlidou, M.; Artero, V. Electrocatalytic Hydrogen Evolution with a Cobalt Complex Bearing Pendant Proton Relays: Acid Strength and Applied Potential Govern Mechanism and Stability. *J. Am. Chem. Soc.* **2020**, *142*, 274-282.

(10) Wang, Y.-H.; Schneider, P. E.; Goldsmith, Z. K.; Mondal, B.; Hammes-Schiffer, S.; Stahl, S. S. Brønsted Acid Scaling Relationships Enable Control Over Product Selectivity from O_2 Reduction with a Mononuclear Cobalt Porphyrin Catalyst. *ACS Cent. Sci.* **2019**, *5*, 1024-1034.
